# Aldehyde Dehydrogenases: Not Just Markers, but Functional Regulators of Stem Cells

**DOI:** 10.1155/2019/3904645

**Published:** 2019-01-13

**Authors:** Giuseppe Vassalli

**Affiliations:** ^1^Laboratory of Cellular and Molecular Cardiology, Cardiocentro Ticino, Lugano, Switzerland; ^2^Faculty of Biomedical Sciences, Università della Svizzera Italiana (USI), Lugano, Switzerland; ^3^Center for Molecular Cardiology, University of Zürich, Zürich, Switzerland

## Abstract

Aldehyde dehydrogenase (ALDH) is a superfamily of enzymes that detoxify a variety of endogenous and exogenous aldehydes and are required for the biosynthesis of retinoic acid (RA) and other molecular regulators of cellular function. Over the past decade, high ALDH activity has been increasingly used as a selectable marker for normal cell populations enriched in stem and progenitor cells, as well as for cell populations from cancer tissues enriched in tumor-initiating stem-like cells. Mounting evidence suggests that ALDH not only may be used as a marker for stem cells but also may well regulate cellular functions related to self-renewal, expansion, differentiation, and resistance to drugs and radiation. ALDH exerts its functional actions partly through RA biosynthesis, as all-trans RA reverses the functional effects of pharmacological inhibition or genetic suppression of ALDH activity in many cell types in vitro. There is substantial evidence to suggest that the role of ALDH as a stem cell marker comes down to the specific isoform(s) expressed in a particular tissue. Much emphasis has been placed on the ALDH1A1 and ALDH1A3 members of the ALDH1 family of cytosolic enzymes required for RA biosynthesis. ALDH1A1 and ALDH1A3 regulate cellular function in both normal stem cells and tumor-initiating stem-like cells, promoting tumor growth and resistance to drugs and radiation. An improved understanding of the molecular mechanisms by which ALDH regulates cellular function will likely open new avenues in many fields, especially in tissue regeneration and oncology.

## 1. Introduction

Stem cells can be defined as cells that undergo symmetric and asymmetric divisions to self-renew or differentiate into mature progeny that can repopulate specific tissues and organs [1, 2]. A more stringent definition requires that the self-renewing ability of stem cells is maintained over the full lifetime of an organism. However, many “stem cell” populations described in the literature actually do not meet the more stringent definition. It has been hypothesized that stem cells in different tissues use common molecular mechanisms to self-renew and differentiate. Hence, common molecular markers shared by stem cells across tissues have been searched for [3]. Three independent large-scale gene array analyses identified putative “stemness” genes in embryonic stem cells (ESCs), hematopoietic stem cells (HSCs), or neural stem cells (NCSs) [4–6]. The finding that only one “stemness” gene (integrin *α*6) was commonly identified in the three studies questioned the existence of a universal molecular signature of stem cells [6]. Rather, a specific type of stem cell may express certain “stemness” genes in a species-specific manner but only a subset of these genes are shared with other stem cell populations. Other characteristic features of stem cells include the absence of markers of cell lineage differentiation (Lin^−^) [7], the ability to efflux Hoechst 33342, and multidrug resistance. The ability to efflux Hoechst 33342, which defines the so-called side population (SP), is related to the expression of the ABC transporter Bcrp1/ABCG2 and other molecular transporters that mediate drug resistance. SP cells from various types of tissues have been associated with self-renewal and multilineage differentiation capacity [8, 9]. However, the metabolic and proliferative state of the cell may influence the SP phenotype. As an example, quiescent populations of self-renewing neural progenitor cells (NPCs) efflux Hoechst 33342 and are identified as SP cells, whereas not actively dividing embryonic NSCs do not efflux Hoechst 33342, despite the fact that they express high levels of ABCG2 [10].

A metabolic stem cell marker that has been described is aldehyde dehydrogenase (ALDH) [11]. By oxidizing a wide range of endogenous and exogenous aldehydes to their corresponding carboxylic acids, this critically important class of NAD(P)^+^-dependent enzymes protect living organisms against oxidative stress. In addition, ALDH plays important roles in the biosynthesis of molecules that regulate cellular homeostasis, such as retinoic acid (RA), *γ*-aminobutyric acid, and betaine [12, 13]. Many ALDH enzymes in evolutionarily distant species protect cells by detoxifying endogenous aldehydes that are formed during lipid peroxidation. ALDH upregulation occurs in bacteria in response to environmental and chemical stressors; in plants in response to dehydration, salinity, and oxidative stress; in yeasts after exposure to ethanol and oxidative stress; and in mammals in response to oxidative stress and lipid peroxidation [14].

High ALDH enzyme activity and a relationship with cyclophosphamide resistance were first described in human HSCs [15]. The development of the Aldefluor™ flow cytometry assay that enables sorting of live ALDH-bright (ALDH*^br^*) cells with high ALDH enzyme activity has been instrumental in moving forward this research area [16, 17]. ALDH*^br^* populations derived from various types of tissues are enriched in self-renewing cells endowed with multilineage differentiation potential. As an example, a few ALDH*^br^* pluripotential cells are able to generate all somatic and reproductive cell lineages in tunicates [18]. In addition, ALDH*^br^* populations from multiple types of cancers are enriched in cells with stem-like characteristics and tumor-initiating ability [19, 20]. However, ALDH*^br^* populations described in the literature typically are heterogeneous, being enriched in, but not consisting exclusively of, stem cells. Many ALDH*^br^* populations that have been reported actually comprised true stem cells, transit amplifying progenitor cells, differentiating progenitors, and even mature cells. It should be noted that the term “ALDH*^br^*” (or ALDH*^high^*) refers to the most highly fluorescent-labeled subset of cells, as assessed with the Aldefluor™ reagent, within a cell population. In contrast, the term “ALDH^+^” is ambiguous and should be avoided, as normal tissues commonly express ALDH. In this review of the literature, the term “ALDH^+^” is used when discussing selected reports that applied this terminology.

The ALDH superfamily is comprised of 19 human isozymes subdivided in 11 families and 4 subfamilies. Many of the ALDH isozymes overlap in relation to subcellular localization (cytoplasm, mitochondria, or nucleus), tissue distribution, and substrate specificities but vary in their efficiency in metabolizing specific aldehydes [12, 13]. In relation to stem cells, much emphasis has been placed on members of the ALDH1 family of cytosolic enzymes required for the biosynthesis of RA, particularly ALDH1A1 and ALDH1A3. This review is aimed at summarizing current evidence suggesting that ALDH not only may be used as a marker of stem cells but also may well regulate cellular function.

## 2. ALDH as a Marker for Normal Stem Cells

Early studies revealed that ALDH*^br^* subpopulations from human umbilical cord blood [17, 21–26], bone marrow [27, 28], and cytokine-mobilized peripheral blood [29, 30] are highly enriched in lineage-committed hematopoietic progenitor cells (HPCs). The ALDH*^br^* subset of cord blood cells includes all long-term and most of the short-term cells that reconstitute hematopoiesis in xenograft models of cord blood transplantation. Retrospective analyses revealed an inverse relationship between the dose of ALDH*^br^* cells administered to patients and the hematopoietic engraftment time [29–31]. A prospective analysis showed a strong direct correlation between ALDH*^br^* cells and colony-forming unit potency of cord blood [32]. While the CD34^+^ subset of ALDH*^br^* bone marrow cells comprises hematopoietic cells, approximately half of ALDH*^br^* bone marrow cells do not express CD34 and are highly enriched for multipotent mesenchymal stem cells (MSCs) and endothelial progenitor cells (EPCs) [27, 28, 33]. ALDH*^br^* cells from bone marrow or cord blood express genes involved in angiogenesis, display proangiogenic activities [34, 35] and promote tissue repair in animal models of limb ischemia [28] and myocardial infarction [36]. In an initial clinical trial in patients with peripheral artery disease, however, autologous ALDH*^br^* bone marrow cell administration failed to improve limb perfusion and functional outcomes [37]. More encouraging results were reported in an early trial in patients with ischemic heart failure [38]. ALDH*^br^* cells in nonmobilized human peripheral blood mainly consist of EPCs and average 0.07% of total white blood cells. The number of circulating ALDH*^br^* cells is inversely correlated with patient age and the severity of coronary artery disease [39].

Regarding the central nervous system, ALDH*^br^* multipotent NPCs have been identified in the developing rat embryonic neural tube [40], fetal mouse brain [41], and both subventricular and subcortical zones of the adult mouse brain [42]. ALDH*^br^* adult brain NPCs can be induced to form neurons, astrocytes, and glia in vitro. When cultured under conditions that facilitate the formation of motor neurons and transplanted i.t. in mouse models of spinal motor atrophy, both ALDH*^br^*-derived neurons and uncultured ALDH*^br^* cells migrated to ventral horns, established functional neuromuscular junctions, and attenuated the progression of the disease [43, 44].

The ALDH*^br^* subpopulation of human skeletal muscle cells is enriched in myoblast progenitors that engraft much more effectively than ALDH*^dim^* cells upon transplantation into skeletal muscle in vivo [45, 46]. Differentiated ALDH*^br^* myoblasts, but not ALDH*^dim^* cells, survive treatment with H_2_O_2_ and diethylaminobenzaldehyde (DEAB), a specific inhibitor of ALDH, in vitro.

The ALDH*^br^* population from human breast reduction specimens is enriched in multipotential cells that give rise to uncommitted, myoepithelial, lumenal epithelial and mixed colonies, as well as ducts upon transplantation into mammary fat pads in vivo. In contrast, ALDH*^dim^* breast cells give rise to lumenal epithelial cells only [47].

ALDH*^br^* central acinar/terminal duct cells from peripheral acinar duct units of the adult mouse pancreas express early embryonic pancreas markers and give rise to endocrine cells with glucose-regulated insulin secretion. When transplanted into mouse embryos, ALDH*^br^* central acinar/terminal duct cells, but not their ALDH*^dim^* counterparts, contribute to both exocrine and endocrine lineages in the developing pancreas [48]. It also has been shown that the ALDH*^br^* subpopulation of human fetal pancreatic cells contains a heterogeneous population enriched in CD133^+^ cells and a subset of markers associated with *β*-cells in the developing human pancreas [49].

**Figure 1:**
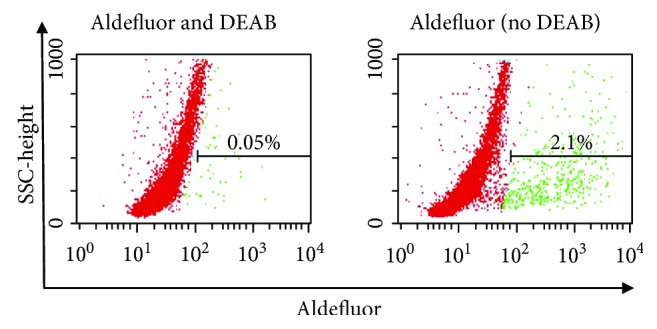
Flow cytometry analysis of ALDH activity in cells isolated from a human cardiac atrial appendage tissue specimen using the Aldefluor™ assay. (a) ALDH*^br^* gating is established by incubating Aldefluor™-reacted cells with the ALDH inhibitor DEAB (negative control). An ALDH/side scatter (SSC) plot is shown. ALDH*^br^* gating was set to include the top 0.05% of DEAB-treated cells with respect to the intensity of the fluorescent signal. (b) Aldefluor-reacted cells analyzed in the absence of DEAB treatment.

ALDH*^br^* progenitors from prostatic epithelial cells express stem cell antigen-1 (Sca-1) and generate prostatic tissue much more effectively than their ALDH*^dim^* counterparts do [50]. The ALDH*^br^* subset of murine nonparenchymal liver cells gives rise to functional hepatocyte-like cells that secrete albumin and urea and display cytochrome P450 activity [51]. The ALDH*^br^*CD44^+^ subset of human skin keratinocytes is enriched in epidermal stem cells with long-term repopulating ability [52]. Data from our laboratory and other groups indicate that ALDH*^br^*CD34^+^ cells from human cardiac atrial appendage tissues (Figure 1) generate mature cardiac myocytes in vitro and in vivo [53, 54]. These findings support the notion that ALDH*^br^* populations from a variety of normal tissues are enriched in stem and progenitor cells.

## 3. ALDH as a Marker of Cancer Stem-Like Cells

Over the past decade, investigation of ALDH expression by stem cells has been focused on so-called cancer stem-like cells (CSCs) [55, 56]. First described in leukemia [57], CSCs were subsequently identified in breast cancer [58] and other solid tumors. In various types of cancers, ALDH*^br^* cells display stem-like features, such as self-renewal, clonogenic growth, tumor-initiating capacity, and drug resistance. Whereas the traditional “stochastic model” of cancer development implies that all cells within a tumor have an equal chance of acquiring mutations and initiating cancer, the alternative “hierarchical model” postulates that only a small subpopulation of tumor cells (i.e., CSCs) are highly efficient at initiating tumors while most tumor cells are differentiated and do not initiate cancer. Designed based on the “stochastic model” of cancer development, most current chemotherapeutics are aimed at shrinking the bulk of solid tumors before surgery and at eradicating cancer cells and metastases that may remain after surgery. Cytotoxic chemotherapeutics typically target proliferating cells; however, CSCs generally are in a quiescent state of the cell cycle [55]. This may be one of the reasons why these treatments fail to cure cancer in many patients. Another reason is expression of ABC transporters by CSCs leading to drug resistance [56]. There is substantial clinical evidence in the literature to suggest that the remaining tumor tissues that survive chemotherapy contain higher numbers of CSCs, including ALDH*^br^* cells, in comparison to the original tumor tissues [59–65]. However, the hypothesis that tumor relapse occurs due to CSCs remains controversial.

The role of ALDH as a potential marker for CSCs has been discussed in depth by previous reviews of the literature [20, 66–69]. ALDH*^br^* cells with tumor-initiating ability have been identified in many types of tissues including breast [47, 60, 61, 70–74], liver [75], colon [59, 76, 77], lung [78, 79], prostate [65, 80, 81], pancreas [62–64, 82], ovary [83], esophagus [84], stomach [85, 86], bone [87], brain [88], skin [89], and bone marrow [90–92]. Particularly instructive are comparative data on ALDH*^br^* and ALDH*^dim^* subsets within a whole tumor cell population showing that ALDH*^br^* cells are more clonogenic and tumorigenic than ALDH*^dim^* cells are [93]. Clinically, the presence of ALDH*^br^* cells has been associated with poorer outcomes in multiple types of cancers [47, 64, 65, 70, 79, 83–86, 90–92, 94, 95]; however, only a few studies identified ALDH*^br^* cells as an independent prognostic factor [47, 70, 95]. Data on ALDH*^br^* cells in malignant melanoma are mixed [96, 97]. Biopsies from patients with metastatic melanoma contain large ALDH*^br^* populations. On the other hand, both ALDH*^br^* and ALDH*^dim^* cells exhibit a comparable aggressive behavior and resistance to antimelanoma drugs [96].

## 4. ALDH in Drug Resistance and Radioresistance

ALDH expression by tumor cells imparts resistance to cytotoxic drugs, such as cyclophosphamide and its active derivative hydroperoxycyclophosphamide (4-HC) [98, 99], doxorubicin [100], cisplatin [101], arabinofuranosyl cytidine (Ara-C) [102], and dacarbazine [96], among others. Cancer cells expressing high levels of ALDH can develop drug resistance of the ALDH-specific activity that catabolyzes a particular drug through oxidation of the specific aldehyde group of the drug [102]. ALDH-dependent drug resistance likewise occurs in normal stem cells. Treatment with chemical inhibitors of ALDH activity sensitizes normal human HSCs and HPCs to the alkylating agent mafosfamide [103], and murine HPCs to 4-HC [104], a clinically used drug for ex vivo purging of residual cancer cells and treatment with autologous bone marrow transplantation [105]. In vitro treatment with interleukin-1 (IL-1) and tumor necrosis factor-*α* (TNF-*α*) stimulates ALDH1 expression and protects human HPCs against 4-HC toxicity. Inhibition of ALDH through DEAB reverses the effect of IL-1 and TNF-*α*, consistent with functional relevance of ALDH activity [106].

Cancer cells with high ALDH activity and other stem-like features have been implicated in drug resistance and tumor relapse; however, this hypothesis remains a matter of debate, as mentioned above. Patients with mantle cell lymphoma (MCL) often respond to initial cytotoxic treatment but subsequently relapse, which is suggestive of the emergence of a clonogenic population of resistant cells. A small population of relatively quiescent, highly clonogenic ALDH^+^ cells resistant to a wide range of chemotherapeutics has actually been identified in MCL patients [107]. Similarly, high-grade serous epithelial ovarian cancer generally responds well to the initial platinum-based chemotherapy but relapses as a result of drug resistance. Treatment with inhibitors of ALDH activity sensitizes drug-resistant ALDH*^br^* ovarian CSCs to chemotherapy [108].

Data from over 100 breast cancer patients who underwent neoadjuvant chemotherapy with paclitaxel and epirubicin suggest that ALDH1^+^ cells, but not CD44^+^CD24^−^ cells, contribute to resistance against chemotherapy [61]. It also has been shown that ALDH1^+^ breast cancers are characterized by negative estrogen receptor (ER^−^), positive human epidermal growth factor receptor type 2 (EGFRII^+^), high expression of the proliferation marker Ki-67, and significantly lower pathologic complete response rates to neoadjuvant chemotherapy compared to ALDH^−^ breast cancers. This study showed that ALDH1, ER, and Ki-67 status are mutually independent predictors of responses to paclitaxel and epirubicin [109]. ALDH*^br^* endothelial cells resistant to fluorouracil in vitro and in vivo have been described in tumor vessels [110].

High ALDH activity has also been implicated in the phenomenon of cellular resistance against radiation. It has been shown that the HER2^+^CD44^+^CD24^−/lo^ subpopulation of breast cancer cells displays high ALDH activity, in vivo tumorigenic potential, and radioresistance [111]. Suppressing ALDH activity through disulfiram prevents the reexpression of stemness genes and the appearance of stem-like properties in breast cancer cells after radiation [112]. A similar relationship between high ALDH activity and radioresistance has been described in prostate cancer progenitor cells [113]. Moreover, the ALDH1 gene is significantly upregulated in the radiation complete response group of human cervical cancer specimens compared to the nonresponsive group. Pretreatment with RA induces the death receptor TRAIL and sensitizes cervical cancer to radiation [114]. Suppressing ALDH1A1 through specific siRNA in murine HSCs induces the expansion of HSCs with radioprotective properties [115]. Collectively, these findings support a central role for ALDH in cellular resistance against chemotherapy and radiation therapy. The underlying mechanism is incompletely understood, but RA biosynthesis and scavenging of reactive oxygen species (ROS) and toxic aldehydes are likely involved [14, 20, 116].

## 5. ALDH and RA Biosynthesis

Members of the cytosolic ALDH1 family are the primary enzymes involved in the biosynthesis of RA, which comprises the most biologically potent retinoid all-trans RA (ATRA), 9-cis-RA, and 13-cis-RA. Retinol (vitamin A) is first oxidized by alcohol dehydrogenase (ADH) to retinaldehyde in a reversible reaction. Retinaldehyde is then metabolized to RA by specific ALDH isozymes (ALDH1A1, ALDH1A2, ALDH1A3, and ALDH8A1) in a tightly regulated, irreversible reaction [12, 117, 118]. Binding of RA with the nuclear RA receptor (RAR; *α*, *β*, or *γ* isoforms)/retinoid X receptor (RXR; *α*, *β*, or *γ* isoforms) heterodimer to the regulatory region (i.e., the RA response element (RARE)) of retinoid-responsive genes triggers transcriptional activation. RA regulates a variety of physiological processes in embryonic development and adult tissue remodeling [119–121]. Chemical inhibition of ALDH through DEAB leads to a decrease in RAR-mediated signaling, delayed cytokine-mediated cell differentiation, and expansion of the most primitive human HSCs in culture. Treatment with ATRA reverses these changes induced by DEAB. These results suggest that ALDH may regulate HSC fate through RA synthesis and that inhibition of ALDH may promote HSC self-renewal through a decrease in RA activity [122].

The regulatory role of retinoid signaling in breast CSC differentiation has been investigated by treating different breast cancer cell lines with DEAB or ATRA and by measuring the respective gene expression profiles. Cells treated with DEAB overexpress genes enriched in a pre-defined breast CSC signature [70], whereas cells treated with ATRA underexpress these genes and contain less CSCs but larger proportions of differentiated cells [123]. ATRA-mediated induction of differentiation in leukemic stem cells has been exploited clinically for the treatment of acute promyelocytic leukemia [124]. Similarly, treatment with RA promotes the differentiation of glioblastoma CSCs and reduces the glioblastoma CSC pool. Constitutive activation of Notch signaling, a downstream target of retinoids, by the Notch intracellular domain abrogates the effect of RA on glioblastoma CSCs [125]. Moreover, treatment with RA activates the phosphatidylinositol-3-kinase (PI3K) signaling pathway through activation of RARs in SH-SY5Y neuroblastoma cells. Activation of PI3K is required for RA-mediated differentiation of neuroblastoma cells [126]. ATRA has also been shown to interact directly with protein kinase C (PKC) [127]. Moreover, RARs and RXRs can form heterodimers with other receptors including ER*α* [128, 129] and peroxisome proliferator-activated receptors (PPAR) *β*/*δ* [130]. Cooperative interaction between RAR*α* and ER*α* is reflected by the requirement for RAR*α* for efficient ER*α*-mediated transcription and cell proliferation, as well as by its relationship with tamoxifen resistance in breast cancer [128, 129]. In ALDH*^br^* multiple myeloma cells, ALDH1A1 activates RXR*α*, which upregulates NIMA-related kinase 2 (NEK2), thereby activating the drug efflux pump and inducing resistance against bortezomib and doxorubicin [131]. These findings suggest that ALDH may promote drug resistance through retinoid signaling.

## 6. ALDH and Oxidative Stress

ROS are constantly produced in living organisms. The inability to handle ROS burden boosts oxidative stress leading to modification of proteins and DNA. Oxidative stress triggers lipid peroxidation of cellular phospholipids yielding over 200 species of reactive aldehydes (e.g., 4-hydroxynonenal (4-HNE)). By metabolizing a wide range of aldehydes, ALDH can attenuate oxidative stress [14]. For example, ALDH2 and ALDH1A1 catabolize 4-HNE to 4-hydroxynon-2-enoic acid [132]. The finding that ALDH*^br^* human skeletal myoblasts, but not ALDH*^dim^* myoblasts, resist toxic effects of H_2_O_2_ in vitro suggests that ALDH activity may impart protection against oxidative stress [46]. CSCs often display lower oxidative stress than differentiated tumor cells, in part due to aberrant cell metabolism of the latter [133]. ALDH*^br^* cells from ovarian clear cell carcinoma exhibit CSC features, contain lower levels of ROS in comparison to ALDH*^dim^* cells, and display upregulation of Nrf2, a key transcriptional factor of the antioxidant scavenger system [134]. ALDH*^br^* Hodgkin lymphoma cells likewise contain lower levels of ROS compared to their ALDH*^dim^* counterparts [135]. Pharmacologic inhibition of ALDH in an epigenetically determined reversibly drug-tolerant subpopulation of cancer cells with CSC characteristics led to the accumulation of ROS to toxic levels specifically in the drug-tolerant subpopulation, suggesting ALDH-mediated protection against oxidative stress in this subpopulation [136]. Inhibition of ALDH in breast cancer cells is associated with increased levels of ROS and downregulation of hypoxia-inducible factor- (HIF-) 2*α*. Treatment with the ROS scavenger N-acetylcysteine abrogates HIF-2*α* downregulation caused by ALDH inhibition. This observation suggests that ALDH activity may stimulate stem cell properties in breast cancer cells through activation of HIF-2*α* [137]. Because chemotherapeutics and radiation produce elevated levels of oxidative stress in cancer cells as part of their beneficial effects, the “ROS scavenging” activity of ALDH could protect cancer cells against these therapeutic approaches by maintaining ROS at low levels [138].

## 7. Regulation of ALDH1A1 and ALDH1A3 Gene Expression and Activity

**Figure 2:**
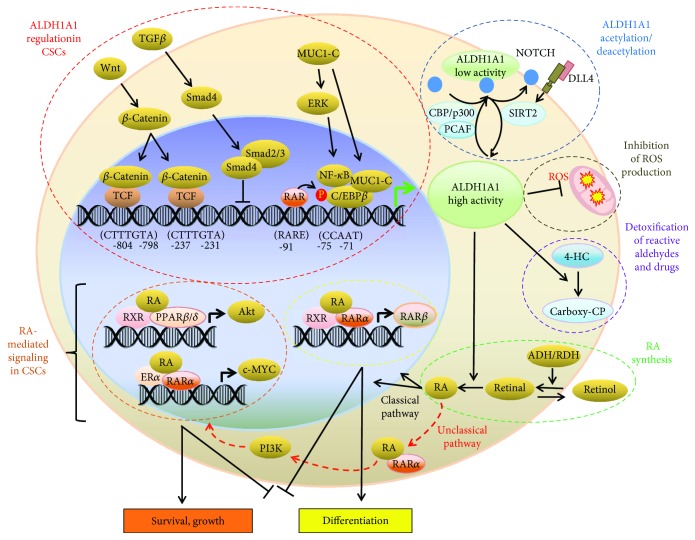
Model of ALDH1A1 regulation, potential retinoid signaling pathways, and functional effects of ALDH in CSCs. Retinol is oxidized to retinal by retinol dehydrogenases, and retinal is then oxidized to RA by ALDH1 enzymes (green dotted line). In the classic pathway, RA enters the nucleus and binds to dimers of RAR*α* and RXRs triggering the expression of its downstream target genes including RAR*β* (yellow dotted line). In the nonclassic pathway, RA binds to dimers of RXRs and PPAR*β*/*δ* to induce the expression of its downstream target genes including Akt (orange dotted line). In cells expressing ER*α*, RA can bind to dimers of RXRs and ER*α* (not shown). RA can also bind with RAR*α* outside the nucleus to activate the PI3K/Akt pathway. Wnt pathway regulates ALDH1A1 through *β*-catenin/TCF-dependent transcription. MUC1-C induces ERK signaling and phosphorylates C/EBP*β*. The complex of MUC1-C and C/EBP*β* occupies the sequence upstream from the transcription initiation site of ALDH1, triggering ALDH1A1 expression. TGF-*β*-induced Smad4 downregulates ALDH1 (red dotted line). Notch promotes ALDH activity in CSCs through induction of deacetylase SIRT2, leading to ALDH1A1 deacetylation, while ALDH1A1 acetylation by acetyltransferase PCAF inhibits ALDH activity (blue dotted line; some parts of this figure were adapted with modifications from Figures 1 and 2 from Xu et al. [20], with editor's permission).

The molecular mechanisms underlying the regulation of ALDH1A1 gene expression are only partly understood. These mechanisms are schematically depicted in Figure 2. Several putative regulatory elements, putative response elements for IL-6 and other molecules, a noncanonical TATA box (ATAAA), and a CCAAT box in the 5′-flanking region of the human ALDH1 gene have been identified. Functional characterization of this region revealed that the CCAAT box region is the main *cis*-acting element that mediates basal ALDH1 promoter activity in Hep3B cells, which express ALDH1, but not in erythroleukemic K562 cells or in fibroblast LTK-cells, which do not express ALDH1. This observation may be related to the finding that cell type-specific factors regulate ALDH1 gene expression [139]. The oncogenic subunit of mucin 1 (MUC1-C), which is aberrantly overexpressed in many human breast cancers, activates ERK signaling and the CCAAT/enhancer-binding protein *β* (CEBP*β*) transcription factor in breast cancer cells. By forming a complex on the ALDH1A1 promoter, MUC1-C and CEBP*β* activate ALDH1A1 gene transcription. These findings suggest the existence of a MUC1-C → ERK → CEBP*β* → ALDH1A1 pathway in breast cancer [140]. Moreover, the *β*-catenin/T-cell factor (TCF) transcriptional complex directly regulates ALDH1A1 gene expression and is associated with radioresistance in prostate cancer progenitors. Suppressing *β*-catenin through specific siRNA reduces the ALDH^+^ population and sensitizes it to radiation. These results support the existence of a *β*-catenin/TCF → ALDH1A1 pathway in prostate cancer progenitors [113]. In these cells, ALDH1 expression is inversely correlated with expression of Smad4, a major mediator of transforming growth factor- (TGF-) *β* signaling. Suppressing Smad4 through specific RNAi upregulates ALDH1A1 mRNA expression whereas Smad4 overexpression downregulates ALDH1A1 mRNA expression in pancreatic adenocarcinoma cells. Negative regulation of ALDH1A1 mRNA transcription by TGF-*β* through binding of Smad4 to the regulatory sequence of the ALDH1A1 gene reduces both the ALDH*^br^* population and the tumor-initiating activity of pancreatic cancer cells [141]. In murine HPCs, Tlx1/Hox11, an oncogenic transcription factor involved in human T-cell leukemia, transcriptionally regulates ALDH1A1 gene expression. ALDH1A1 overexpression alters murine hematopoiesis, favoring myeloid differentiation over lymphopoiesis [142].

The regulation of ALDH1A3 gene has been investigated in the chemoresistant subpopulation of malignant pleural mesothelioma cells, which express ALDH1A3 as the main ALDH isozyme responsible for ALDH activity in these cells. Suppressing ALDH1A3 through RNAi prevents the survival of ALDH*^br^* malignant pleural mesothelioma cells, especially after treatment with chemotherapeutics. Binding of CEBP*β* to the ALDH1A3 promoter activates ALDH1A3 gene expression in these cells. CEBP*β* forms a complex with DDIT3/CHOP/GADD153. STAT3-NF*κ*B activity represses DDIT3 mRNA leading to CEBP*β*-dependent ALDH1A3 promoter activation. Conversely, inhibition of STAT3-NF*κ*B activity increases DDIT3 expression and DDIT3-CEBP*β* complex formation, which reduces the occupancy of the ALDH1A3 promoter by CEBP*β* and ALDH1A3 expression, resulting in an increase in pemetrexed or cisplatin-induced ALDH*^br^* cell death [143]. In A549 lung alveolar adenocarcinoma cells, the Oct4 paralog Oct1, a transcription factor associated with the expression of stem cell markers, positively regulates the ALDH*^br^* subpopulation [144].

Mounting evidence suggests that epigenetic regulation of stem cell-related genes is involved in drug resistance [145]. The bromodomain and extraterminal (BET) family of proteins play an important role in this regard. BET proteins including BRD4 recognize acetylated lysine on histones through their bromodomains and control transcription of their target genes either directly by recruiting the transcriptional machinery or indirectly through enhancer elements. Genes hypersensitive to inhibition by BET proteins typically exhibit BRD4 occupancy at superenhancer elements [146]. BRD4 targets the promoters of stem cell-related genes [147]. It has been shown that a clinically applicable small-molecule inhibitor of BET suppresses ALDH activity by abrogating BRD4-mediated ALDH1A1 expression through a superenhancer element and its associated enhancer RNA (eRNA) in ovarian cancer cells [148]. The formation of chromatin looping that allows enhancer and promoter interaction is a significant part of enhancer function [149]. Direct examination of chromatin looping between the superenhancer and *ALDH1A1* gene promoter using chromosome conformation capture (3C) in cells with or without treatment with the BET inhibitor revealed that the BET inhibitor abrogates the chromatin looping between the superenhancer and the promoter of the *ALDH1A1* gene. These findings support a role for BRD4 in the transcriptional control of the *ALDH1A1* gene through regulation of its superenhancer and the associated eRNA. The BET inhibitor suppresses the outgrowth of ovarian cancer cells treated with cisplatin *in vitro* and *in vivo* while also improving the survival of ovarian cancer bearing mice treated with cisplatin [148].

Posttranslational regulatory mechanisms of ALDH activity have also been described. The acetylation state of lysine 353 (K353), which is modulated by acetyltransferase P300/CBP-associated factor (PCAF) and deacetylase sirtuin 2 (SIRT2), regulates ALDH1A1 activity. ALDH1*^br^* human breast cancer cells with low levels of ALDH1A1 acetylation display self-renewal characteristics. In contrast, high levels of ALDH1A1 acetylation are associated with a depletion of the stem cell population and decreased self-renewal. Notch induces ALDH1A1 deacetylation through SIRT2 activation, thereby promoting ALDH activity and stem-like cellular features [150]. Stimulation with sphingosine-1-phosphate (S1P) expands the ALDH1^+^ subpopulation of ER^+^ MCF-7 breast cancer cells which display CSC features. S1P binding to the S1P receptor 3 (S1PR3) stimulates ligand-independent Notch activation. Overexpression of sphingosine kinase 1 induces an increase in the levels of S1P and an expansion of the CSC pool [151]. Similar correlations between Notch signaling, ALDH activity, and CSCs have been observed in murine osteosarcoma [152] and non-small cell lung cancer. Chemical or genetic suppression of the Notch pathway decreases the number of ALDH^+^ cells and the clonogenic potency of lung adenocarcinoma cells [94]. A549 lung cancer cells express both ALDH1A1 and ALDH3A1. Incubation of these cells with ATRA or other commercially available retinoids decreases protein levels and enzyme activity of ALDH1A1 and ALDH3A1, but not the corresponding mRNAs, and increases cytotoxicity of 4-HC and acetaldehyde. These findings suggest a posttranslational mechanism by which retinoids may decrease the expression of both ALDH1A1 and ALDH3A1 [153]. Collectively, these reports illustrate several molecular mechanisms that regulate ALDH gene expression and enzyme activity.

## 8. Functional Roles of ALDH1A1 and ALDH1A3 in Normal Stem and Progenitor Cells

ALDH isozymes are widely expressed across tissues but regulate cellular function in a species- and tissue-specific manner [12, 13]. The ALDH inhibitor DEAB used as a negative control in the Aldefluor™ assay was originally viewed as a specific inhibitor of the ALDH1 family; hence, ALDH enzyme activity measured with this assay was assumed to reflect the expression of ALDH1 isozymes [154]. However, recent data indicate that DEAB is not a specific inhibitor when assayed in vitro versus ALDH1, ALDH2, and ALDH3 family members [155]. Several studies have correlated ALDH*^br^* cells, as assessed with the Aldefluor™ reagent, and cells staining positive with ALDH1-targeting antibodies, in various tissues and cell populations, such as human breast epithelium [47] and MSCs [156]. However, many reports lacked clarification of isozyme specificity due to limited information about the isozyme specificity of both the Aldefluor™ assay and ALDH-targeting antibodies [66].

Gene expression profiling has revealed higher expression levels of ALDH1A1 in normal human HSCs [157] and in normal mouse HSCs [158, 159] in comparison to less primitive hematopoietic cells. Suppressing ALDH1A1 through specific siRNA delays RA-mediated differentiation of murine HSCs, resulting in the expansion of primitive cells with radioprotective features [115]. Of note, ALDH1A1 deficiency does not alter ALDH activity nor does it affect HSC and NSC functions in the ALDH1A1^−/−^ mouse [160]. This finding suggests that other ALDH isozymes may compensate for the loss of ALDH1A1 function in the ALDH1A1^−/−^ mouse.

ALDH1A3 is the most highly expressed ALDH gene in normal human mammary tissue. In contrast, ALDH1A1 is expressed at low levels in all mammary epithelial cells. While the most primitive human mammary stem cells with bilineage differentiation potential are associated with low ALDH activity, marked upregulation of ALDH1A3 expression and ALDH activity occurs at the point of commitment to the lumenal lineage [161]. We recently measured mRNA expression of all 19 human ALDH isozymes and identified ALDH1A3 as the most highly expressed ALDH isozyme in human cardiac-derived progenitor cells. ALDH1A3 expression was confirmed at the protein level. Suppressing ALDH1A3 (but not ALDH1A1, ALDH1A2, ALDH2A, ALDH4A1, or ALDH8A1) through specific siRNA markedly decreased ALDH activity and cell proliferation. ALDH*^br^* cells exhibited higher propensity for differentiating into mature cardiac myocytes, as compared to ALDH*^dim^* cells [53]. Another study demonstrated high expression levels of RNA-binding protein Sam68 (Khdrbs1) in neurogenic areas of the neocortex. Khdrbs1 regulates ALDH1A3 pre-mRNA 3′-end processing and promotes self-renewal in mouse embryonic NPCs. The Khdrbs1*^−/−^* mouse displays decreases in ALDH1A3 expression and ALDH enzyme activity in NPCs, a depleted embryonic NPC pool, and reduced cortical expansion [162]. Taken together, these results document functional roles of ALDH1A1 and ALDH1A3 in normal stem and progenitor cells.

## 9. Functional Roles of ALDH1A1 and ALDH1A3 in CSCs

It has been proposed that the role of ALDH as a CSC marker comes down to the specific isoform(s) expressed [163]. The investigational focus has been on the ALDH1 family owing to its role in RA biosynthesis and drug resistance. Expression of ALDH1A1 by CSCs has been demonstrated in multiple types of cancers [164], such as human breast cancer [47], lung cancer [94], and colon cancer. Suppressing ALDH1A1 through specific siRNA sensitizes colon cancer cells to chemotherapy [165]. The expression of ALDH1A2 [101, 155, 166], ALDH1A3 [71, 72, 74, 77, 89, 167–171], ALDH1B1 [76, 172], ALDH2 [155], ALDH3A1 [80, 173–176], ALDH3B1 [177, 178], ALDH4A1 [179], ALDH5A1 [180–183], ALDH6A1 [184], ALDH7A1 [185, 186], and ALDH10 [187] has been reported in various malignancies.

A recent study showed that the majority of small-sized Ki-67^+^ proliferating progenitors within human HCC1937 breast cancer cells selectively express high levels of ALDH1A3 [74]. Analyses of breast patient tumors revealed that high levels of ALDH1A3 are correlated with the expression of RA-inducible genes containing RAREs, poorer patient survival, and triple-negative breast cancers. Both ALDH1A3 and treatment with ATRA promoted the expression of RA-inducible genes in MDA-MB-231, MDA-MB-468, and MDA-MB-435 breast cancer cell lines. ALDH1A3 had opposing effects in tumor xenografts, increasing tumor growth and metastasis of MDA-MB-231 and MDA-MB-435 cells but decreasing them in MDA-MB-468 cells. Treatment with ATRA replaced ALDH1A3 in inducing the same opposing tumor growth and metastasis effects, suggesting that ALDH1A3 exerted these effects through activation of RA signaling. ALDH1A3 induced largely divergent gene expression profiles in MDA-MB-231 and MDA-MB-468 cells, in line with the opposing tumor growth effects. Treatment with the DNA methylation inhibitor 5-aza-2′-deoxycytidine restored RA inducibility of RARE-containing HOXA1 and MUC4 in MDA-MB-231 and MDA-MB-468 cells. These results indicate that differences in epigenetic modifications contribute to differential ALDH1A3/RA-induced gene expression, thereby influencing tumor growth and metastasis in breast cancer [72].

Recent data suggest that expression of ALDH1A3 may affect in vitro proliferation and invasion of colon cancer cells dependent on the CXCR4 status [77]. In patients with high-grade glioma, two subtypes of glioma CSCs have been described: proneural CSC and mesenchymal glioma CSCs. The latter are maintained by activated glycolytic metabolism involving ALDH1A3 and display a more aggressive behavior than proneural CSCs. Inhibition of ALDH1A3 selectively suppresses the growth of mesenchymal glioma CSCs [188]. In mesenchymal glioma CSCs, ALDH1A3 has been shown to regulate the expression of the survival factor tissue transglutaminase [189]. In neuroblastoma cell lines expressing high levels of ALDH1A3, ALDH1A3 knockout via CRISPR/Cas9 gene editing results in decreased clonogenicity of tumor-initiating cells [88]. In melanoma cells, ALDH1A3 is upregulated through epigenetic mechanisms, as compared to normal melanocytes. Melanoma treatment with a novel irreversible isoform-specific ALDH1 inhibitor (DIMATE) or depletion of ALDH1A1 and/or ALDH1A3 results in the accumulation of toxic aldehydes, increased apoptosis, and decreased tumor growth in xenograft mouse models [190].

As to the role of ALDH isozymes in mediating drug resistance, ALDH1A1, ALDH1A3, and ALDH3A1 are known to convert active 4-HC to inactive carboxyphosphamide [15, 104]. De novo expression of ALDH1 through transfection of cell lines induces resistance against anti-cancer alkylating agents in hamster V79 cell lines. This effect is completely reversed by pretreatment with DEAB [191]. Overexpression of ALDH1 using a retroviral vector imparts 4-HC resistance to human HPCs in vitro and after bone marrow transplantation in vivo [192]. High ALDH3A1 expression and activity correlate with cell proliferation and increased tolerance to the cytostatic and cytotoxic effects of lipidic aldehydes [176].

Taken together, these findings indicate that ALDH1A1, ALDH1A3, and ALDH3A1 exert important functions in CSCs. Functional roles of other ALDH isozymes in CSCs remain to be elucidated.

## 10. ALDH and Other Stem Cell Markers

Various combinations of markers including ALDH, CD34, CD133, Sca-1, CD44, and integrin *α*2*β*1 have been used to identify putative stem cells, as exemplified by ALDH*^br^*CD34^+^ human cardiac atrial stem cells [53, 54], ALDH*^br^*CD133^+^ human fetal pancreatic stem cells [49], ALDH*^br^*Sca-1^+^ murine prostatic stem cells [50], and ALDH*^br^*CD44^+^ human epidermal stem/progenitor cells [52]. It is worth noting that, besides ALDH, other stem cell-associated markers, such as CD133 and CD44, may also play functional roles in stem cells [193, 194]. Unfortunately, only a limited number of comparative marker analyses are available. A prospective study identified ALDH, but not CD34, as a predictor of colony-forming unit potency of cord blood [32]. Another report showed lack of correlation of stem cell markers in breast cancer stem cells. ALDH activity and other commonly used stem cell markers including CD24, CD44, SOX2 expression, mammosphere-forming ability, and the SP phenotype were variably present in populations from human breast cancer tissue and breast cancer cell lines and identified cancer cell subpopulations with no reliable therapeutic implications [73]. Further comparative studies of ALDH and other stem cell markers are warranted. Although ALDH may represent a superior stem cell marker for selected applications, such as cord blood transplantation, and a useful marker of CSCs in multiple types of malignancies, it cannot be viewed as the best universal stem cell marker within all biological contexts.

## 11. Conclusions

Both normal stem cells and CSCs are typically characterized by increased ALDH enzyme activity with concomitant upregulation of specific ALDH isozymes, primarily ALDH1A1, ALDH1A3, and ALDH3A1. Suppressing ALDH activity by knocking down ALDH1A1 or ALDH1A3 results in a depletion of the stem cell pool while also sensitizing stem cells to chemotherapy in multiple types of tissues. ALDH regulates cellular function primarily through retinoid signaling and detoxification of a variety of aldehydes. Molecular mechanisms that regulate ALDH expression and activity have been described. Unlike cell surface antigens, ALDH represents an intracellular, metabolic marker. In analogy to the former, however, ALDH shows limited specificity for true stem cells. As an example, the most primitive human mammary stem cells with bilineage differentiation potential display low ALDH activity whereas mammary stem cells at the point of commitment to the lumenal lineage exhibit highest ALDH activity [161], as mentioned above. ALDH*^br^* transient amplifying progenitors, and even ALDH*^br^* differentiated cells, such as tumor endothelial cells, have been described [110]. Measuring specific ALDH isozymes expressed by a particular type of stem or progenitor cell will likely improve the sensitivity and specificity of the markers and, most important, pinpoint novel therapeutic targets. In this regard, ALDH1A subfamily-selective inhibitors [195] including clinically applicable small-molecule inhibitors [148] have been developed. An improved understanding of the functional roles specific ALDH isozymes play in stem cell biology will help design novel strategies for regenerative medicine and in the fight against cancer.
